# Ablation of Mouse Selenium-Binding Protein 1 and 2 Elevates LDL by Disruption of Cholesterol Efflux and Lipid Metabolism †

**DOI:** 10.3390/ijms26073363

**Published:** 2025-04-03

**Authors:** Shuangli Zhao, Yingxia Song, Yuko Nakashima, Xing Zou, Takayuki Koga, Takumi Ishida, Renshi Li, Yuko Hirota, Yoshitaka Tanaka, Yuji Ishii

**Affiliations:** 1 Division of Pharmaceutical Cell Biology, Graduate School of Pharmaceutical Sciences, Kyushu University, Fukuoka 812-8582, Japan; zhao.shuangli.783@s.kyushu-u.ac.jp (S.Z.); songyingxia410@outlook.com (Y.S.); zouxing_hbtcm@163.com (X.Z.); yhirota@phar.kyushu-u.ac.jp (Y.H.); ytanaka@phar.kyushu-u.ac.jp (Y.T.); 2 Laboratory of Hygienic Chemistry, Daiichi University of Pharmacy, Fukuoka 815-8511, Japan; koga@daiichi-cps.ac.jp; 3 School of Pharmacy, International University of Health and Welfare Fukuoka, Ohkawa 831-8501, Japan; ishida@iuhw.ac.jp; 4 School of Traditional Chinese Pharmacy, China Pharmaceutical University, Nanjing 211198, China; li-renshi@cpu.edu.cn

**Keywords:** PPARs, cholesterol/efflux, lipids/oxidation, triglycerides, liver

## Abstract

Selenium-binding protein 1 (SeBP1) is an anticancer factor that affects lipid metabolism in mouse kidneys via the peroxisome proliferator-activated receptor-alpha (PPARA) pathway. However, its physiological role in the liver is difficult to explain because of the presence of the highly homologous selenium-binding protein 2 (SeBP2). To investigate the role of these proteins in the liver, we generated *SeBP1* and *SeBP2* double-knockout mice (*SeBP1/2*-DK). *SeBP1/2* deletion did not significantly alter the mice phenotypic compared to that of the wild-type strain. Then, we identified the genes involved in hepatic lipid metabolism. The double knockout did not affect fatty acid and cholesterol synthesis, but inhibited fatty acid oxidation and cholesterol efflux. Furthermore, transfection of HepG2 cells with human selenium-binding protein 1 (hSeBP1) positively regulated PPARA and the genes controlled by it. Overexpression of hSeBP1 reduced the levels of non-esterified fatty acids in the culture medium. The serum levels of low-density lipoprotein cholesterol, high-density lipoprotein cholesterol, and triglycerides were significantly different among the three groups. In summary, we elucidated the potential signaling pathways of SeBP1 and SeBP2 in fatty acid oxidation and hepatic cholesterol efflux. Our findings provide insights relevant for developing new strategies to prevent and treat lipid metabolism disorders.

## 1. Introduction

Selenium is a vital trace element that contributes significantly to liver health by being a key component of selenoproteins [1]. These proteins possess strong antioxidant and anti-inflammatory abilities, aiding in the reduction of oxidative stress [2] and inflammation [3], which are critical factors contributing to liver damage [4]. Due to the extensive health benefits of selenium, there has been a growing focus on selenium supplementation [5].

Currently, 25 and 24 selenoprotein genes have been identified in the human and mice genome, respectively [6,7]. Mouse selenium-binding protein 1 (SeBP1) is a homolog of human SELENBP1 and encodes a protein of approximately 56 kDa containing a critical selenium-binding domain in its molecular structure [8,9,10]. This structural characteristic enables SeBP1 to interact with selenium, thereby mediating its metabolism and transport within cells [11]. SeBP1 influences the activity of various selenium-dependent enzymes, such as glutathione peroxidase 1 and glutathione peroxidase 4, consequently affecting intracellular redox balance and antioxidant responses [12,13]. Decreased SeBP1 expression, correlating with aberrant cell metabolism associated with tumor development and progression, has been reported in multiple cancers [14,15,16,17].

In a previous study, we observed that SeBP1 expression in the liver was significantly induced by dioxin-like compounds and 2,3,7,8-tetrachlorodibenzo-*p*-dioxin (TCDD) in rats and mice [18,19]. Therefore, SeBP1 may be involved in the toxicity caused by TCDD. It is also possible that the induction of SeBP1 expression is a response mechanism for the mitigation of this toxicity. However, the minimal difference in TCDD toxicity between *SeBP1* single-knockout mice (*SeBP1-*SK) and WT mice is speculated to result from the compensatory effect of selenium-binding protein 2 (SeBP2) on SeBP1 function [19]. SeBP2 shares 98% homology with SeBP1 and functions as an acetaminophen-binding protein [20]. This isoform is present in mice but not in humans and rats [20]. To reduce the effects of SeBP2 and clarify the role of SeBP1, in previous studies, our group subjected *SeBP1-*SK mice to 20 h fasting and found significant reduction in *SeBP2* mRNA levels in kidneys. We proposed that the ablation of *SeBP1* affects lipid metabolism via the peroxisome proliferator-activated receptor-alpha (PPARA) pathway [21]. PPARA, a nuclear receptor, forms a heterodimer with the retinoid X receptor alpha (RXRA) and is closely associated with abnormalities in lipid metabolism [22]. The dysregulation of its activity leads to metabolic disorders, such as insulin resistance [23], hyperlipidemia [24], and atherosclerosis [25]. Furthermore, human selenium-binding protein 1 (hSeBP1) has been identified as a methanethiol oxidase that produces H_2_S from methanethiol [26,27]; hSeBP1 is a mature adipocyte marker [28].

Although some progress has been made in SeBP1 research, several aspects related to the physiological functions of this protein need to be clarified. First, will *SeBP1/2* double-knockout mice (*SeBP1/2*-DK) affect the mouse phenotype? Second, what are the potential mechanisms underlying the effects of *SeBP1/2* deletion? Can SeBP1/2 in the liver affect the synthesis and metabolism of fatty acids via the PPARA pathway? If so, what specific mechanisms are involved? Does the deletion of SeBP1/2 affect cholesterol metabolism and efflux? In this study, we aimed to explore and answer these questions using *SeBP1/2*-DK mice.

## 2. Results

### 2.1. Generation of SeBP1/2-DK Mice and Their Phenotypes

**Figure 1:**
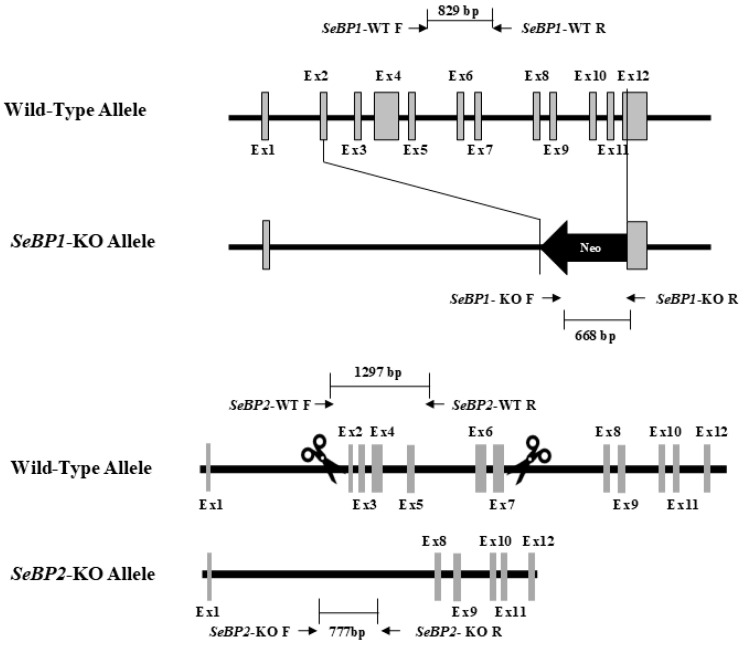
Construction of the targeting allele for knocking out *SeBP1* and *SeBP2*.

**Figure 2:**
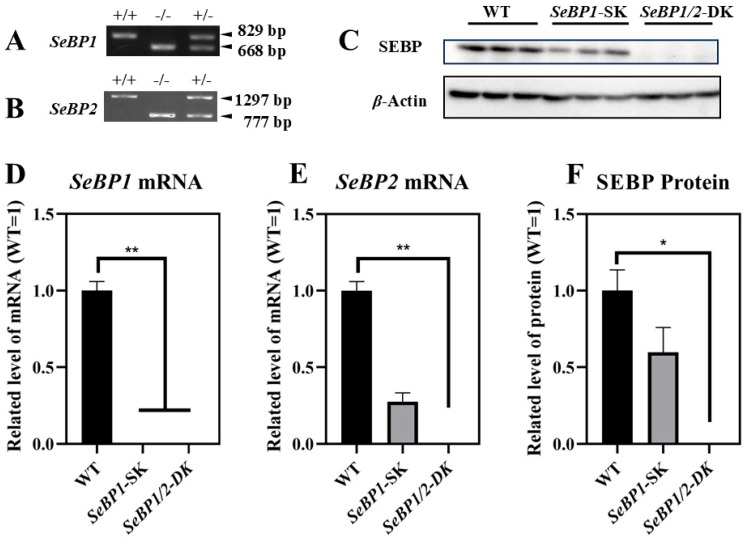
Confirmation of SeBP1 and SeBP2 deletion. SeBP1 (**A**) and SeBP2 (**B**) genotyping. Absence of SeBP1 expression in *SeBP1*-SK and *SeBP1/2*-DK mice (**D**,**E**). Bars represent the mean  ±  SEM for 6 male mice. The absence of SeBP expression was confirmed using Western blotting (**C**,**F**). Bars represent the mean  ±  SEM for 3 male mice. WT, wild type; *SeBP1*-SK, *SeBP1* knockout mice; *SeBP1/2*-DK, *SeBP1* and *SeBP2* double-knockout mice. *, *p* < 0.05; **, *p* < 0.01.

**Figure 3:**
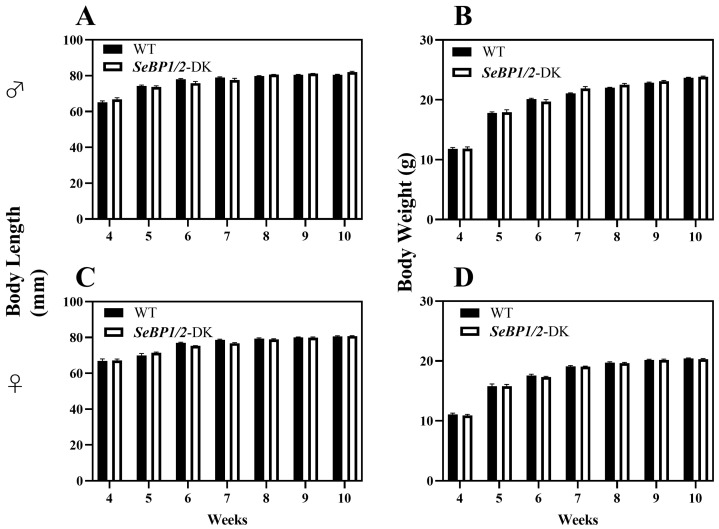
Body length and body weight of male (**A**,**B**) and female (**C**,**D**) *SeBP1/2*-DK and WT mice. Bars represent the mean  ±  SEM for 6 female and 10 male mice. WT, wild type; *SeBP1/2*-DK, *SeBP1* and *SeBP2* double-knockout mice.

**Figure 4:**
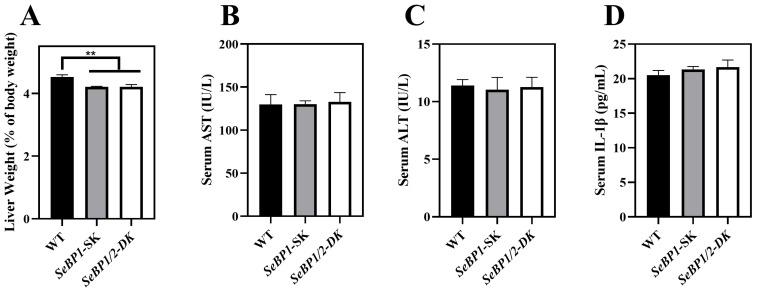
Effect of *SeBP1/2* deletion on liver injury parameters. Relative liver weight (% of body weight) for WT, *SeBP1*-SK, and *SeBP1/2*-DK male mice at 8 weeks (**A**). Serum AST (**B**) and ALT (**C**) activity and IL-1β levels (**D**) in WT, *SeBP1*-SK, and *SeBP1/2*-DK male mice were determined using commercially available kits at 8 weeks. Each bar represents the mean  ±  SEM for 6 samples. WT, wild type; *SeBP1*-SK, *SeBP1* knockout mice; *SeBP1/2*-DK, *SeBP1* and *SeBP2* double-knockout mice. **, *p* < 0.01.

To explore the role of hepatic SeBP in metabolism, we generated *SeBP1/2*-DK mice using a CRISPR/Cas9-mediated gene deletion method, as described for *SeBP1*-SK mice (Figure 1) [18]. In the PCR-based screening of mice, an 829 bp PCR product was obtained for WT mice, whereas a 668 bp product was obtained for *SeBP1*-SK and *SeBP1/2*-DK mice (Figure 2A). Moreover, WT and *SeBP1*-SK mice showed a 1297 bp PCR product, whereas *SeBP1/2*-DK mice showed a 777 bp PCR product (Figure 2B). The mRNA expression levels of *SeBP1* and *SeBP2* supported the successful construction of *SeBP1*-SK and *SeBP1/2*-DK mice (Figure 2D,E). Even though SeBP protein expression was detectable in the liver of *SeBP1*-SK mice, it tended to reduce compared to that in the liver of WT mice. The protein was not detected in the liver of *SeBP1/2*-DK mice (Figure 2C,F). Although no change in body weight or length was noticeable (Figure 3A–D), a significant reduction in the liver weight (% of body weight) compared with that in WT mice was observed (Figure 4A). The weights of other tissues were not altered by knockout of the single or double knockout (Table 1). Therefore, we examined the roles of *SeBP1* and *SeBP2* in the liver.

**Figure 5:**
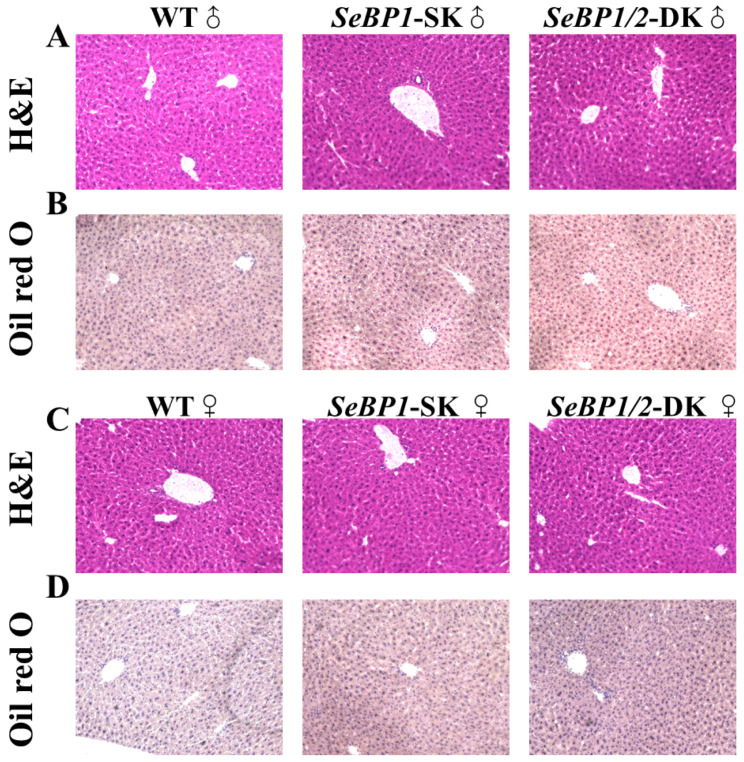
Hematoxylin and eosin (H&E) and oil red O staining of liver tissue sections. H&E staining of the liver tissue sections from male (**A**) and female (**C**) WT, *SeBP1*-SK, and *SeBP1/2*-DK mice at 8 weeks. Oil red O staining of the liver tissue sections from male (**B**) and female (**D**) WT, *SeBP1*-SK, and *SeBP1/2*-DK mice at 8 weeks. The images were acquired using a Keyence BZ-X800 microscope (20×). WT, wild type; *SeBP1*-SK, *SeBP1* knockout mice; *SeBP1/2*-DK, *SeBP1* and *SeBP2* double-knockout mice.

**Figure 6:**
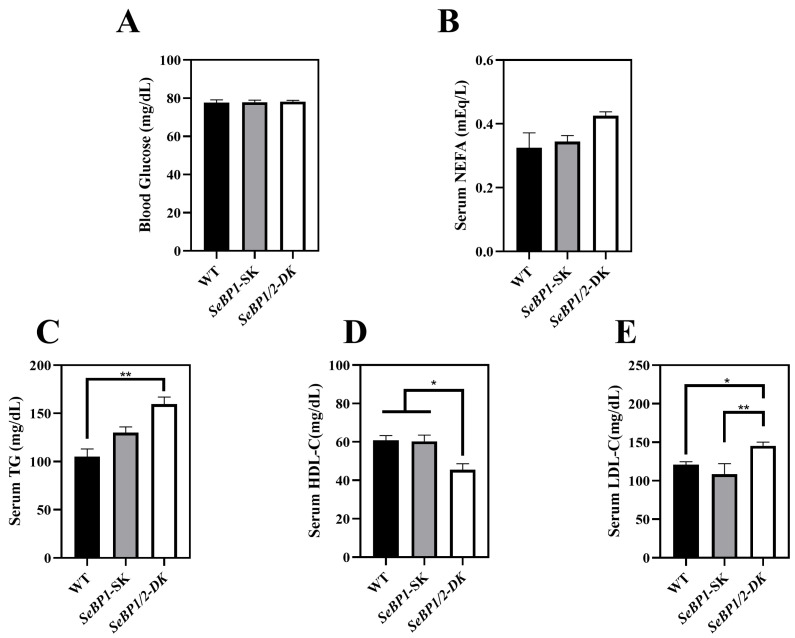
Serum parameters in 8 weeks-old WT, *SeBP1*-SK, and *SeBP1/2*-DK male mice. Each bar represents the mean  ±  SEM for 6 samples. The level of blood glucose (**A**), serum NEFA (**B**), TG (**C**), HDL-C (**D**), and LDL-C (**E**) levels were determined using commercially available kits. WT, wild type; *SeBP1*-SK, *SeBP1* knockout mice; *SeBP1/2*-DK, *SeBP1* and *SeBP2* double-knockout mice; NEFA, non-esterified fatty acids; TG, triglycerides; HDL-C, high-density lipoprotein cholesterol; LDL-C, low-density lipoprotein cholesterol. *, *p* < 0.05; **, *p* < 0.01.

Considering the fact that aspartate aminotransferase (AST) and alanine aminotransferase (ALT) levels increase after myocardial infarction or liver disease [29], we analyzed the levels of these proteins to check for acute or chronic liver injury. No significant differences were observed among the three groups (Figure 4B,C). The results of serum IL1-β levels (Figure 4D) as well as H&E staining (Figure 5A,C) also indicated that the *SeBP1* or *SeBP1/2* deletion did not result in a direct liver injury. In addition, the results of oil red O staining (Figure 5B,D) showed that the double knockout did not cause significant lipid accumulation. We found that the deletion of *SeBP1* and *SeBP2* did not affect the level of blood glucose (Figure 6A) and serum NEFA (Figure 6B); However, serum TG levels were significantly increased (Figure 6C). Compared to the WT group, statistically significant differences were noted in HDL-C and LDL-C levels among the three groups (Figure 6D,E). These results indicate that *SeBP1* and *SeBP2* play important roles in lipid metabolism.

### 2.2. Deletion of SeBP1/2 Affects β-Oxidation of Fatty Acids in Mice Liver

**Figure 7:**
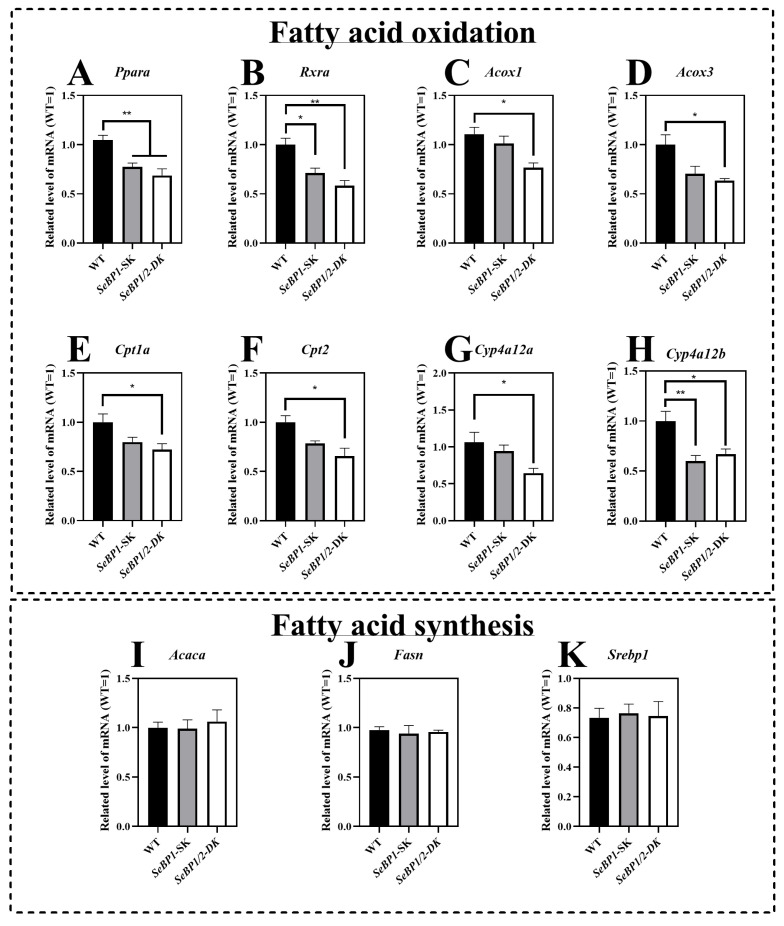
*SeBP1/2* affects key genes associated with β-oxidation of fatty acids in the liver. WT, *SeBP1*-SK, and *SeBP1/2*-DK male mice were fasted for 20 h at 8-weeks of age and euthanized by CO_2_ inhalation. The livers were snap-frozen and stored at −80 °C. Total RNA was isolated from the liver using an RNeasy Mini Kit (Qiagen, GmbH, Hilden, Germany). Values represent the means ± SEM for 5 mice. *Ppara*, peroxisome proliferator-activated receptor alpha; *Rxra*, retinoid X receptor alpha; *Acox1*, acyl-CoA oxidase 1; *Acox3*, acyl-CoA oxidase 3; *Cpt1a*, carnitine palmitoyl transferase 1A; *Cpt2*, carnitine palmitoyl transferase 2; *Cyp4a12a*, cytochrome P450, family 4, subfamily a, polypeptide 12A; *Cyp4a12b*, cytochrome P450, family 4, subfamily a, polypeptide 12B; *Acaca*, acetyl-coA carboxylase alpha; *Fasn*, fatty acid synthase; *Srebp1*, sterol regulatory element-binding protein 1; WT, wild type; *SeBP1*-SK, *SeBP1* knockout mice; *SeBP1/2*-DK, *SeBP1* and *SeBP2* double-knockout mice. *, *p* < 0.05; **, *p* < 0.01.

The mRNA expression of *Ppara* and *Rxra* was significantly reduced in the *SeBP1/2*-DK and *SeBP1*-SK groups (Figure 7A,B). To further clarify the relationship between SeBP and lipid metabolism, we investigated the expression of *Ppara*-related target genes. We observed that the mRNA levels of acyl-coenzyme A oxidase 1 (*Acox1*) and acyl-coenzyme A oxidase 3 (*Acox3*) were significantly reduced upon simultaneous deletion of *SeBP1* and *SeBP2* (Figure 7C,D). Furthermore, *SeBP1* deletion did not significantly alter the mRNA levels of carnitine palmitoyl transferase 1a (*Cpt1a*) and carnitine palmitoyl transferase 2 (*Cpt2*). Surprisingly, in the *SeBP1/2*-DK group, the expression of *Cpt1a* and *Cpt2* was significantly reduced (Figure 7E,F). Additionally, members of the cytochrome P450 (Cyp)4a subfamily, *Cyp4a12a* and *Cyp4a12b*, which are involved in the ω-oxidation of fatty acids, were also significantly downregulated in the *SeBP1/2*-DK group (Figure 7G,H). All these results indicate that the deletion of *SeBP1/2* may affect peroxisomal and mitochondrial β-oxidation of free fatty acids. However, no significant effect on fatty acid synthesis was observed (Figure 7I–K).

### 2.3. Overexpression of hSeBP1 in HepG2 Promotes β-Oxidation of Fatty Acids

**Figure 8:**
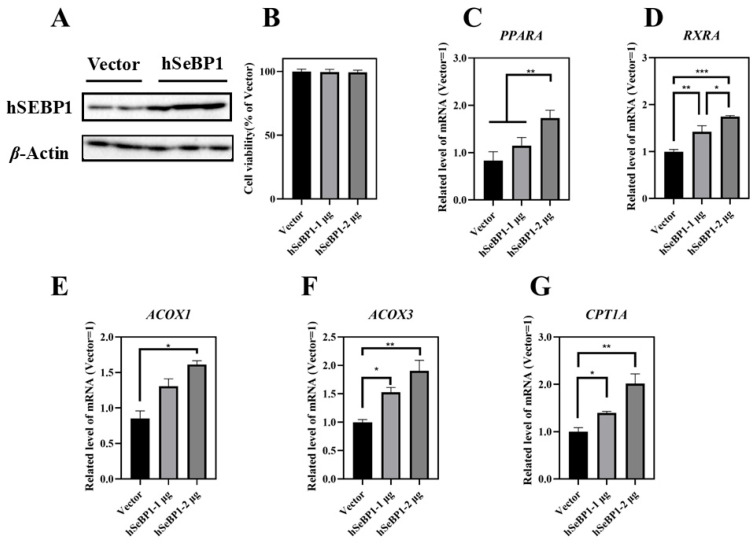
Effect of hSeBP1 transfection on key genes associated with oxidation of fatty acids in HepG2 cells. Each bar represents the mean  ±  SEM for 5 samples. HepG2 cells were seeded in 6-well plates and transfected with pcDNA3.1-hSeBP1 (1 or 2 µg/well); the empty pcDNA-3.1(-)-hygro vector was used for the control group. (**A**) Western blot analysis of hSEBP1 expression in HepG2 cells. (**B**) Effects of hSeBP1 overexpression on the viability of HepG2 cells. (**C**–**G**) mRNA level of *PPARA*, *RXRA*, *ACOX1*, *ACOX3,* and *CTP1A* in HepG2 cells. *PPARA*, peroxisome proliferator-activated receptor alpha; *RXRA*, retinoid X receptor alpha; *ACOX1*, acyl-CoA oxidase 1; *ACOX3*, acyl-CoA oxidase 3; *CPT1A*, carnitine palmitoyl transferase 1A. *, *p* < 0.05; **, *p* < 0.01; ***, *p* < 0.001.

**Figure 9:**
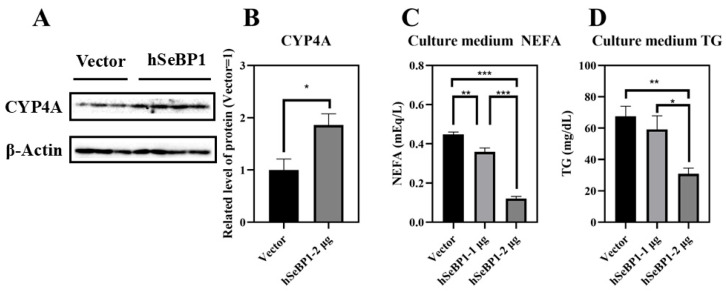
Effect of hSeBP1 overexpression on CYP4A, NEFA, and TG levels. Each bar represents the mean  ±  SEM for 3 or 4 samples. HepG2 cells were seeded in 6-well plates and transfected with pcDNA3.1-hSeBP1 (1 or 2 µg/well); the empty pcDNA-3.1(-)-hygro vector was used for the control group. NEFA and TG levels in culture medium were determined using commercially available kits. (**A**,**B**) Western blot analysis of CYP4A expression in HepG2 cells. The level of culture medium NEFA (**C**) and TG (**D**) levels was determined using commercially available kits. *, *p* < 0.05; **, *p* < 0.01; ***, *p* < 0.001.

To investigate the effect of hSeBP1 on fatty acid metabolism, hSeBP1 was overexpressed in HepG2 hepatocellular carcinoma cells. The expression of hSeBP1 was significantly increased after transfection (Figure 8A). The results of MTT assay indicated that the transfection did not affect the viability of these cells (Figure 8B). Overexpression of hSeBP1 promoted the expression of *PPARA* (Figure 8C) and its transcriptional targets, *ACOX1*, *ACOX3*, and *CPT1A* (Figure 8E–G). These results suggest that hSeBP1 not only catalyzes the conversion of acyl-CoA to enoyl-CoA, but also facilitates the entry of long-chain acyl-coenzyme A into the mitochondria via CPT1A. Intriguingly, hSeBP1 overexpression in HepG2 also significantly improved the protein level of CYP4A (Figure 9A,B), which is a downstream gene of PPARA. Furthermore, we examined the concentration of NEFA in the medium to investigate the effect of SeBP1 on the degree of β-oxidation of fatty acids. Surprisingly, the concentrations of NEFA and TG in the medium were significantly reduced upon transfection in a concentration-dependent manner (Figure 9C,D). Overall, these results support the notion that hSeBP1 may positively regulate β-oxidation of fatty acids.

### 2.4. Knockout of SeBP1/2 Inhibits Cholesterol Transport from the Liver but Has No Effect on Cholesterol Synthesis in Mice

**Figure 10:**
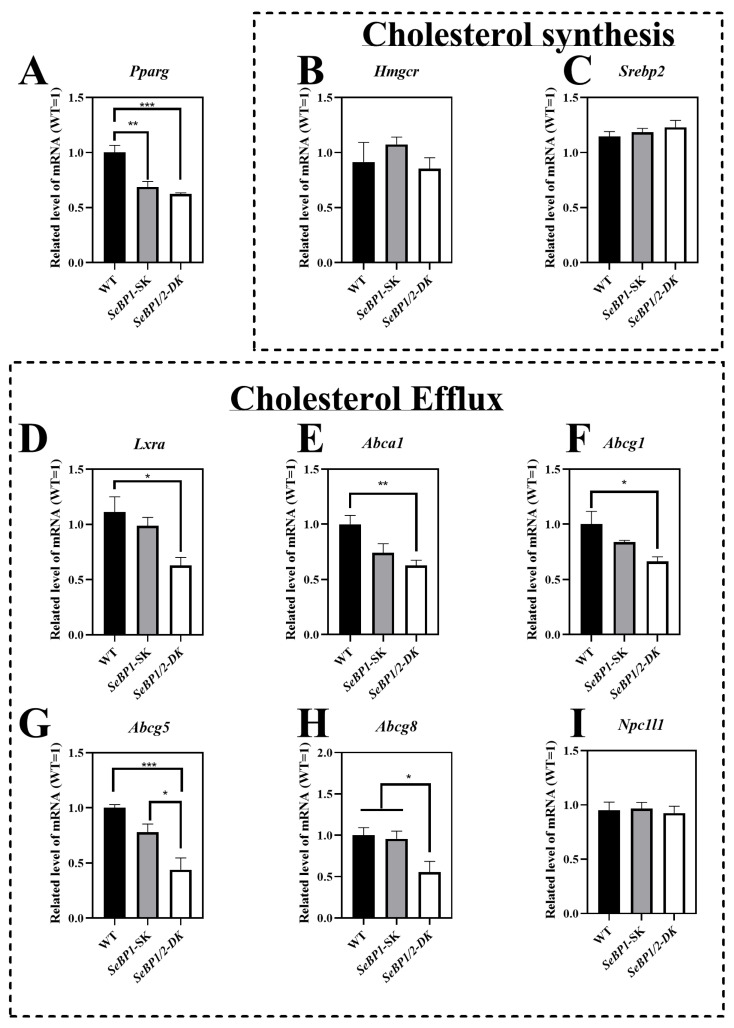
SeBP1 and SeBP2 affect key genes associated with cholesterol transport from liver. Each bar represents the mean  ±  SEM for 6 male mice. *Pparg*, peroxisome proliferator-activated receptor gamma; *Hmgcr,* 3-hydroxy-3-methylglutaryl-CoA reductase; *Srebp2*, sterol regulatory element-binding protein 2; *Lxra*, liver X receptor alpha; *Abca1*, ATP-binding cassette transporter A1; *Abcg1*, ATP-binding cassette transporter G1; *Abcg5*, ATP-binding cassette transporter G5; *Abcg8*, ATP-binding cassette transporter G8; *Npc1l1*, Niemann–Pick c1-like 1; WT, wild type; *SeBP1*-SK, *SeBP1* knockout mice; *SeBP1/2*-DK, *SeBP1* and *SeBP2* double-knockout mice. *, *p* < 0.05; **, *p* < 0.01; ***, *p* < 0.001.

The LDL-C, HDL-C, and TG levels exhibited statistically significant differences among the three groups. Given that the liver is the primary organ for cholesterol production and is responsible for most of the cholesterol synthesis in the body, we analyzed the expression of key enzymes involved in the endogenous synthesis pathway of cholesterol synthesis in the liver. We did not find any significant differences among the three groups (Figure 10B,C). The mRNA levels of *Pparg* (Figure 10A), *Lxra* (Figure 10D), *Abca1*(Figure 10E), *Abcg1*(Figure 10F), *Abcg5*(Figure 10G), and *Abcg8* (Figure 10H) were significantly reduced in the *SeBP1/2*-DK group. These results indicate that the double knockout may inhibit hepatic cholesterol efflux, but does not significantly affect cholesterol synthesis.

### 2.5. SeBP1 and SeBP2 Are Positive Regulators of Superoxide Dismutase In Vivo and In Vitro

**Figure 11:**
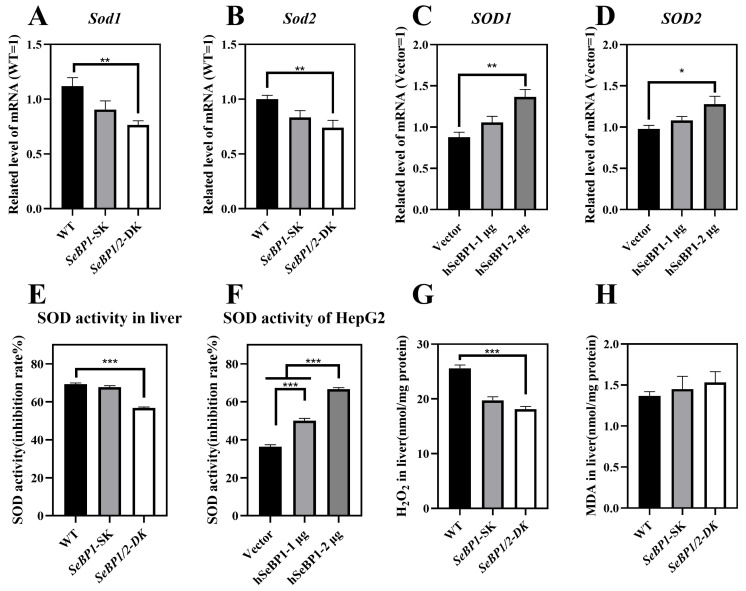
SeBP1 and SeBP2 are positive regulators of superoxide dismutase in vivo (8 weeks-old male mice) and in vitro. (**A**,**B**) mRNA level of *Sod1*, and *Sod2* in the liver of male mice. (**C**,**D**) mRNA level of *SOD1*, and *SOD2* in HepG2 cells. The hepatic SOD activity in liver (**E**) or HepG2 (**F**) was determined using a commercially available kit. (**G**) The concentration of hydrogen peroxide in the liver of mice was measured using a quantitative peroxide assay. (**H**) Thiobarbituric acid-reactive substance (TBARS), which represents the predominant malonedialdehyde (MDA) is used as an indicator of hepatic lipid peroxidation in male mice. Each bar represents the mean  ±  SEM for 6 samples. *SOD1*, superoxide dismutase 1; *SOD2*, superoxide dismutase 2; MDA, malondialdehyde; WT, wild type; *SeBP1*-SK, *SeBP1* knockout mice; *SeBP1/2*-DK, *SeBP1* and *SeBP2* double-knockout mice. *, *p* < 0.05; **, *p* < 0.01; ***, *p* < 0.001.

We checked whether the reduction in *PPARA* expression affected its downstream genes, *Sod1* and *Sod2*, which encode major cytoplasmic antioxidant enzymes [30,31]. We determined the expression and activity of superoxide dismutase (SOD) in mice and in the in vitro overexpression model. We analyzed the mRNA levels of *Sod1* and *Sod2* in the liver. As shown in Figure 11A,B, the expression of both these genes was drastically reduced. The expression of *Sod1* and *Sod2* in mice liver was positively regulated by SeBP. Compared with that in the WT group, the SOD activity showed no change in the *SeBP1*-SK group but was significantly lower in the *SeBP1/2*-DK group (Figure 11E). SOD is an important antioxidant that catalyzes the conversion of superoxide radicals to oxygen and hydrogen peroxide through a disproportionation reaction. Additionally, overexpression of SeBP1 significantly increased the mRNA expression and activity of *SOD1* and *SOD2* (Figure 11C,D), which was also evidenced by the increased SOD activity (Figure 11F). Furthermore, hepatic H_2_O_2_ levels in the *SeBP1/2*-DK group were significantly reduced compared with those in the WT group (Figure 11G). Finally, the *SeBP1/2* deletion tended to increase the malondialdehyde (MDA) levels in the liver (Figure 11H). Collectively, these results suggest that the deletion of *SeBP1/2* effectively inhibits SOD activity and increases oxidative stress in the liver.

## 3. Discussion

In a previous study, our research group showed that SeBP1 affects lipid metabolism in the kidneys of mice via the PPARA pathway [21]. However, its specific mechanism of action has not yet been elucidated. We found that the expression of SeBP2—a homolog of SeBP1 in the liver—was not aborted in the mice liver, the center of lipid metabolism, upon fasting unlike in the kidneys, which showed much lower levels of SeBP2 mRNA, making it very challenging to understand the role of SeBP2 in the liver [21]. Given the lack of clarity regarding the roles of SeBP1 and SeBP2 in the liver, *SeBP1/2*-DK mice were generated in our laboratory for further exploration [32]. We found that the double knockout did not affect the height and weight of female and male offspring (Figure 3A–D), indicating that these deletions did not affect the growth and reproduction of mice. Additionally, it did not affect the coefficients of the major organs (Table 1), except for the liver of male mice (Figure 4A). We further assessed the effects on AST and ALT, which play crucial roles in biochemical processes within the human body, and alterations in their activities are widely employed in the diagnosis of diseases, such as liver and myocardial infarction [29]. Compared with the WT group, we did not find any significant changes in their activity, indicating that the deletion of *SeBP1/2* did not cause acute or chronic liver injury (Figure 4B,C and Figure S1A,B). Moreover, the results of serum IL-1β levels (Figure 4D and Figure S1C) and H&E staining of the liver tissue sections (Figure 5A,C) also revealed the absence of any significant change among the three groups. These results suggest that deletion of *SeBP1/2* caused no serious problem in mice.

Next, we analyzed some biochemical indicators in mice. Deletion of *SeBP1/2* did not alter the blood glucose levels in male mice (Figure 6A and Figure S1D). The liver plays a crucial role in lipid metabolism and is the primary organ responsible for the synthesis, breakdown, storage, and transport of lipids. It assimilates free fatty acids from the bloodstream and converts them into TG for storage and energy production [33]. Additionally, lipoproteins produced by the liver transport cholesterol and TG to the tissues throughout the body for energy use or storage. The liver also regulates the balance between the HDL-C and LDL-C levels to maintain stable lipid levels in blood [34]. Thus, hepatic lipid metabolism is essential for sustaining overall energy balance and metabolic health. Because liver weights were significantly reduced in the *SeBP1/2*-DK group, we next focused on the liver to investigate the function of SeBP1 and 2. Preliminarily, the oil red O staining of the liver tissue sections did not reveal any significant differences among the three groups (Figure 5B,D). We observed significant changes in TG levels in the *SeBP1/2-DK* group compared with those in the WT group (Figure 6C). Although there was no statistically significant change, an upward trend was observed for NEFA levels (Figure 6B). Lipid abnormalities, particularly in fatty acid metabolism, can have major effects on the development of atherosclerosis and other metabolic diseases. Fatty acids are catabolized via β-oxidation, which is crucial for maintaining energy balance [35]. Although the exact mechanism is not clear, we reported that in mouse kidneys, SeBP1 may affect lipid metabolism through the PPARA pathway, but not through peroxisome proliferator-activated receptor-delta (PPARD) or peroxisome proliferator-activated receptor-gamma (PPARG) [21].

In the present study, we found that ablation of *SeBP1/2* downregulated the β-oxidation of fatty acids in liver of mice via the PPARA signaling pathway (Figure 7A,B). Additionally, we examined the expression of genes associated with β-oxidation, particularly *Cpt1a* and *Cpt2*, which are classical targets of PPARA. CPT1A imports long-chain fatty acids into the mitochondria, whereas CPT2 converts acyl-carnitines back into acyl-CoA esters for mitochondrial oxidation. CPT1A catalyzes an important rate-limiting step in mitochondrial fatty acid oxidation, and its deficiency results in reduced β-oxidation of fatty acids [36]. Here, for the first time, we found that SeBP1/2 knockout significantly decreases the mRNA expression of *Cpt1a* and *Cpt2* (Figure 7E,F).

PPARA also plays a key role in regulating enzymes of peroxisomal fatty acid oxidation, including ACOX1 and ACOX3. ACOX is an important enzyme in lipid metabolism, which converts acyl coenzyme A to 2-trans-enoyl coenzyme A for peroxisomal fatty acid β-oxidation [37]. Peroxisome acyl-CoA oxidase 1 (ACOX1) is the primary catalase enzyme in the peroxisomal β-oxidation pathway, the dysfunctioning of which leads to abnormal lipid metabolism and hepatocellular carcinoma, and potentially results in metabolic disorders. ACOX1 mainly oxidizes long-chain and ultra-long-chain fatty acids, whereas its related enzymes, ACOX3, catalyze the oxidation of branched-chain fatty acids. We found that knocking out *SeBP1/2* led to decreased expression of *Acox1*, which indicates that SeBP1 and SeBP2 play a regulatory role in its expression (Figure 7C). As a paralog of ACOX1, ACOX3 plays a crucial role in the β-oxidation of branched-chain fatty acids within peroxisomes. We performed qRT-PCR and found that the expression of *Acox3* decreased after knocking out *SeBP1/2* (Figure 7D). In addition to *Cpt1a*, the *Acox1*, *Acox3*, and several other genes, which are downstream of PPARA were also downregulated in the liver of *SeBP1/2*-DK mice. For example, *Cyp4a12a* and *Cyp4a12b* were downregulated upon *SeBP1/2* deletion (Figure 7G,H). These genes are involved in the ω-oxidation of fatty acids, a process that typically serves as a minor catabolic pathway for medium- to long-chain fatty acids [38]. However, in female mice, we did not observe any significant change in the expression of these genes among the three groups (Figure S2), which suggested that *SeBP1/2* deletion may exert sex-based differences on β-oxidation of fatty acids. Fatty acid transport, esterification, and de novo lipogenesis in the liver may be affected by estrogens [39].

We overexpressed hSeBP1 in HepG2 cells to further elucidate the underlying mechanism [40]. Our findings are consistent with those of previous studies showing that SeBP1 is barely detectable in highly metastatic HepG2 cells [41]. Transfection experiments were successfully performed with HepG2 cells (Figure 8A). The MTT assay results indicated that transfection of HepG2 cells with hSeBP1 (1 or 2 µg) did not affect the cell viability (Figure 8B). Furthermore, the expression of *PPARA*, *RXRA*, *CPT1A*, *ACOX1*, and *ACOX3* was positively correlated with SeBP1 overexpression (Figure 8C–G).

CYP4A is a major fatty acid ω-hydroxylase that catalyzes the hydroxylation of both saturated and unsaturated fatty acids to prevent lipotoxicity [42]. Studies have indicated that lower *Cyp4a* gene expression causes the accumulation of free fatty acids and esterified fatty acids, aggravating hepatic steatosis and inflammation [43]. Conversely, upregulating *Cyp4a* gene expression can accelerate fatty acid oxidation and mitigate hepatic steatosis [38]. Furthermore, *Cyp4a10*, a downstream target gene of PPARA, plays a critical role in the oxidative degradation of hepatic fatty acids and triglycerides [44,45]. Our results showed that overexpression of hSeBP1 promotes the expression of CYP4A (Figure 9A,B), consistent with qRT-PCR results observed in mice (Figure 7G,H). In addition, the NEFA and TG levels in the culture medium were reduced by hSeBP1 overexpression in HepG2 cells (Figure 9C,D). These results indicate that SeBP1 and SeBP2 inhibit the accumulation of lipids in the liver by increasing fatty acid oxidation.

SeBP1 acts as a tumor suppressor gene. SeBP1 expression is significantly reduced in poorly differentiated lung adenocarcinomas compared to that in moderately and well-differentiated lung adenocarcinomas [14]. In addition, in bladder cancer [16], thyroid cancer [17], and hepatocellular carcinoma [41], downregulation of SeBP1 was associated with poor survival. A defining characteristic of cancer cells is that unlike normal cells, they continuously undergo excessive division. Lipid production is crucial not only for the synthesis of DNA and proteins but also for the growth and proliferation of cancer cells. Known for their vital structural roles as key components of cell membranes, lipids contribute significantly to the cell architecture. Moreover, lipids function as signaling molecules in cancer and participate in post-translational modifications of proteins [46]. Presumably, SeBP1 acts as a cancer suppressor by enhancing fatty acid oxidation, reducing the formation of phospholipids, which are cell membrane components, and hindering the formation of cell membranes.

Abnormalities in lipid metabolism, particularly disturbances in cholesterol transport, are key to the onset and progression of atherosclerosis. HDL-C and LDL-C play crucial roles in cholesterol metabolism and cardiovascular health [33]. HDL-C transports excess cholesterol from peripheral tissues and other lipoproteins to the liver, reducing the risk of cholesterol build-up and plaque formation in the arteries. Excessive LDL-C levels lead to cholesterol deposition in the arterial walls, accelerating the onset of atherosclerosis. Many studies have shown a negative correlation between HDL-C levels and the risk of developing atherosclerotic cardiovascular disease [33]. We found that the double knockout significantly reduced the serum HDL-C levels and significantly increased the serum LDL-C levels (Figure 6D,E). HDL-C synthesis is intricately linked to two transmembrane cholesterol proteins, ABCG1 and ABCA1, which are responsible for transporting free cholesterol to mature HDL [47].

We found that the mRNA levels of *Abca1* and *Abcg1* decreased significantly in the *SeBP1/2*-DK group (Figure 10E,F). LXRA, a member of the LXR subfamily, is associated with cholesterol and lipid metabolism, adipogenesis, and carbohydrate metabolism. Substantial evidence suggests that ABCG1 and ABCA1 are at least partially regulated at the transcriptional level by LXRA [48]. Additionally, unsaturated fatty acids inhibit the expression of ABCG1 and ABCA1 by affecting the LXR/RXR response element [49]. In addition, studies have shown that selenium activates the *LXRA*–*ABCA1* pathway, promoting cholesterol efflux from cells [50]. Consistent with these observations, we found that the mRNA levels of *Lxra* in the liver were significantly downregulated in *SeBP1/2*-DK mice (Figure 10D). The mRNA levels of *Abcg5* and *Abcg8*, which are also regulated by LXRA, were significantly downregulated in the SeBP1/2-DK group (Figure 10G,H). PPARG, a key nuclear transcription factor for lipid metabolism, plays an important role in regulating lipid metabolism [51]. A previous study showed that PPARG activators induce ABCA1 expression and cholesterol removal from macrophages [51]. We found that *Pparg* levels were significantly decreased in the liver (Figure 10A). In female mice, although a trend for change in the expression of some genes was noted, no significant differences were observed among the three groups (Figure S3). A previous study showed that estrogen enhances cholesterol efflux pathways [52], whereas males typically exhibit higher LDL-C levels, lower HDL-C levels, and reduced cholesterol efflux efficiency, which predispose them to hypercholesterolemia and cardiovascular disease in early adulthood to during middle age [53].

SOD is a critical antioxidant enzyme that protects cells from oxidative damage by catalyzing the dismutation of superoxide radicals into H_2_O_2_ and O_2_ [54]. We observed that the ablation of *SeBP1/2* led to a decrease in the mRNA levels of *Sod1* and *Sod2* (Figure 11A,B) and effectively inhibited the SOD activity (Figure 11E), which may explain the reduction in H_2_O_2_ levels (Figure 11G). However, the expression of *Sod1* and *Sod2* was apparently not affected by the absence of *SeBP1/2* in female mice (Figure S4). Additionally, a trend of increasing levels of MDA, which serves as an indicator of oxidative stress and indirectly reflects cellular damage, was observed (Figure 11H). The mRNA levels and activities of SOD1 and SOD2 were significantly increased by the overexpression of hSeBP1(Figure 11C,D,F), and ablation of *SeBP1/2* may lead to an increased risk of oxidative stress.

These results suggest that SeBP1/2 may affect not only β-oxidation of fatty acids via PPARA but also cholesterol efflux via PPARG. Selenium, an essential trace element, has several important physiological roles, such as antioxidant activity, reducing the risk of atherosclerosis [1], and preventing cardiovascular diseases [2]. SeBP1, as a selenium-binding protein, exerts a similar effect. Recent studies identified SeBP1 as a potential therapeutic target for aortic stenosis [55], possibly because of its role as a marker of white adipocytes and intracellular lipid accumulation, thereby influencing lipid metabolism [28]. In addition, SeBP1 was found to be associated with adipose maturation, but did not affect lipid synthesis [28], which is consistent with our findings that *SeBP1/2* double knockout did not affect fatty acid and sterol synthesis. In this study, we further clarified the relationship between SeBP1 and lipid metabolism. Moreover, for the first time, we propose that SeBP1 is a potential therapeutic target for managing conditions related to abnormal fatty acid oxidation and cholesterol efflux.

Given that *SeBP1/2* are deleted throughout the organism, their absence could have wide-ranging effects on various tissues, including the adipose tissue and muscle, as well as on the immune system. These changes may indirectly affect liver metabolism, potentially altering lipid storage, inflammatory responses, or oxidative stress pathways. Further studies examining the effects of liver-specific knockout may provide valuable insights into the mechanisms through which these genes regulate hepatic function and contribute to the overall metabolic homeostasis. A high-sugar diet was reported to suppress *SeBP1* expression in the liver [28], and combined with the results of the present study, the use of a model of abnormal lipid metabolism or a high-fat model would be more useful in understanding the role of SeBP1/2 in the liver.

In summary, we generated *SeBP1/2*-DK mice for the first time and used them to explore the roles of SeBP1 in lipid metabolism. We found that ablation of *SeBP1/2* did not affect fertility or growth and did not cause liver injury in mice. Moreover, our results indicate that SeBP1/2 may promote mitochondrial and peroxisomal fatty acid oxidation in the liver, via the PPARA/CPT1A/ACOX1 pathway. Our study is the first to show that SeBP1/2 affects intrahepatic cholesterol efflux via the PPARG/LXRA/ABCA1 pathway. Overall, our findings indicate that ablation of *SeBP1/2* may increase the risk of abnormal fatty acid oxidation and cholesterol efflux. These findings not only contribute to clarifying the physiological function of SeBP but also provide novel therapeutic guidance and insights for managing patients suffering from diseases triggered by abnormal lipid metabolism.

## 4. Materials and Methods

### 4.1. Construction of SeBP1/2 Knockout Mice

Cas9 mRNA was prepared from a linear DNA template using a CAS500A-1 Transcription-Ready Cas9 SmartNuclease mRNA kit (System Biosciences, Palo Alto, CA, USA), according to the manufacturer’s instructions. sgRNA was generated using the CAS510A-1 Linearized T7 gRNA SmartNuclease Vector Kit (System Biosciences, Palo Alto, CA, USA). The sequences for sgRNA synthesis were as follows: sgRNA1: GGTGGAGATCCGCAAGTTCA; sgRNA2: CAGCTTCTGTTTGTCTCGTG. sgRNA1 and sgRNA2 were digested with EcoRI. Cas9 mRNA, sgRNA1, and sgRNA2 were microinjected into fertilized embryos of *SeBP1*-SK mice at a ratio of 2:1:1. *SeBP1*-SK mice were generated as described in our previous study [18]. In the offspring, deletion mutations in exons 2 and 7 of mouse *SeBP2* gene sequence were identified. Ten-week-old *SeBP1/2* double heterozygous knockout mice were produced by Unitech (Chiba, Japan) and inbred to produce the F2 progeny. PCR screening was performed to detect the deletion of the *SeBP1* and *SeBP2* genes in the F2 progeny. The primers used for genotyping are listed in Table S1. The animals were housed in a specific pathogen-free (SPF) facility with a controlled light/dark cycle (light period from 7:00 AM to 7:00 PM), constant temperature (22 ± 5 °C), and constant humidity (50 ± 15%). The animals had free access to standard chow (CE-2; CLEA Japan, Tokyo, Japan) and clean water. Eight-week-old WT, *SeBP1*-SK, and *SeBP1/2*-DK mice were fasted for 20 h and euthanized by CO_2_ inhalation between 9 and 11 a.m. Blood was collected, allowed to stand for 1 h at 25 °C, and then centrifuged at 700× *g* for 15 min at 4 °C to obtain serum. Livers and serum were snap-frozen, and stored at −80 °C. All animal experiments were performed in accordance with the guidelines of the Kyushu University Animal Care and Experimentation Committee.

### 4.2. Cell Culture and Transfection

Human liver cancer cell line HepG2 was purchased from Cellular Engineering Technologies Inc. (Coralville, IA, USA) and cultured in Dulbecco’s modified Eagle medium (High Glucose) (FUJIFILM Wako, Osaka, Japan), supplemented with 10% fetal bovine serum (FBS, Sigma-Aldrich, St. Louis, MO, USA) and 1% penicillin–streptomycin (FUJIFILM Wako, Osaka, Japan) at 37 °C with 5% CO_2_. Cells were seeded in 6-well plates at a density of 2 × 10^5^ cells/well and cultured for 24 h. pcDNA3.1(+)-hSeBP1, containing human SELENBP1 cDNA (GenBank #NM_003944.4) with 3′-insertion of a 24 bp sequence (GATTACAAGGATGACGACGATAAG) encoding the DYKDDDDK-tag, was purchased from GeneScript Japan (Tokyo, Japan). Next, pcDNA3.1-hSeBP1 was transfected into HepG2 cells using 1 mg/mL polyethyleneimine hydrochloride MAX (PEI, linear, molecular weight 40,000, transfection grade) (Polysciences, Inc., Warrington, PA, USA), and the cells were incubated for 48 h. Thereafter, the cells and culture medium were harvested for qRT-PCR, Western blotting, and biochemical analyses.

### 4.3. Cell Viability Assay

Cell viability was assessed using the MTT assay. After the cells were subjected to various treatments in 96-well plates, they were treated with 0.5 mg/mL MTT and incubated at 37 °C for 4 h. Thereafter, the solution in the wells was discarded and 150 μL dimethyl sulfoxide was added to each well. The optical density (OD) was measured at 570 nm using a U-1800 spectrometer (Hitachi Ltd., Tokyo, Japan).

### 4.4. Western Blot Analysis

Liver tissue was collected and homogenized in 0.25 M sucrose solution (pH 7.4) containing cOmplete™ Protease Inhibitor Cocktail and phosSTop™ Protease Inhibitor Cocktail (Roche Diagnostics GmbH, Mannheim, Germany), using a tissue homogenizer. Cultured cells were washed with ice-cold PBS, collected using a cell scraper, and lysed in ice-cold immunoprecipitation buffer containing 20 mM Tris (pH 7.4), 150 mM NaCl, 1% Triton X-100, 10% glycerol, and 1% phenylmethylsulfonyl fluoride for 30 min. Both tissue and cell homogenates were centrifuged at 9000× *g* for 20 min at 4 °C. Protein concentration was determined using the Lowry method [56]. Equal amounts of protein extracts were resolved on sodium dodecyl–polyacrylamide gels and then transferred onto activated polyvinylidene difluoride membranes. The membranes were blocked with 5% skim milk and incubated overnight at 4 °C with primary antibodies against SeBP, CYP4A1/4A2/4A3 (Santa Cruz Biotechnology, Dallas, TX, USA) and β-Actin (BioVision Inc., Mountain View, CA, USA) at a dilution of 1:5000. Subsequently, the membranes were incubated at room temperature for 1 h with horseradish peroxidase-labeled anti-mouse IgG (GE Healthcare, Chicago, IL, USA), diluted 1:5000. Protein signal was detected using Clarity™ Western ECL Substrate and the band densities were quantitated using the Image Lab4.1 software (Bio-Rad, Hercules, CA, USA).

### 4.5. Biochemical Analysis

Serum aspartate aminotransferase (AST), alanine aminotransferase (ALT), low-density lipoprotein cholesterol (LDL-C), high-density lipoprotein cholesterol (HDL-C), triglycerides (TG), and non-esterified fatty acids (NEFA) were quantified using commercial kits (FUJIFILM Wako Co., Osaka, Japan). Glucose levels were analyzed using a blood glucose monitoring system (Jiangsu yuyue Medical Equipment & Supply Co., Ltd. Danyang, China). The superoxide dismutase (SOD) activity assay kit was purchased from Dojindo Laboratories (Kumamoto, Japan). The ELISA kit for serum IL-1β was obtained from BioLegend, Inc. (San Diego, CA, USA). Hydrogen peroxide (H_2_O_2_) levels were determined using the Pierce™ Quantitative Peroxide Assay Kit, which was purchased from Thermo Fisher Scientific Inc. (Waltham, MA, USA).

### 4.6. Lipid Peroxidation

Peroxide levels in the mouse liver were determined using a thiobarbituric acid reaction. 1,1,3,3-Tetramethoxypropane and thiobarbituric acid were acquired from FUJIFILM Wako Co., Ltd. (Osaka, Japan) and Sigma-Aldrich, Inc. (St. Louis, MO, USA), respectively.

### 4.7. Histopathology

The liver tissue was fixed by perfusing with 4% paraformaldehyde solution, embedded in paraffin, and cut into 4 µm-thick sections with a microtome (CM3050S; Leica Microsystems, Wetzlar, Germany) for H&E staining. The sections were stained with hematoxylin and eosin to examine morphological damage. Frozen sections (10 μm thick) were embedded in Tissue-Tek OCT compound (Sakura, Tokyo, Japan) for oil red O staining. The images of the stained sections were acquired using a Keyence BZ-X800 microscope (Keyence, Osaka, Japan).

### 4.8. Real-Time Quantitative Reverse Transcription PCR (qRT-PCR)

The RNeasy Mini Kit (Qiagen, GmbH, Hilden, Germany) was used to isolate total RNA from tissues and cells. The extracted RNA was treated with gEraser to remove any contaminating genomic DNA and subsequently reverse transcribed into cDNA (Takara Bio, Shiga, Japan). Next, target mRNA was amplified using the Fast SYBR Green Master Mix (Thermo Fisher Scientific) on a StepOnePlus Real-Time PCR System (Thermo Fisher Scientific). The sequences of the primers used are listed in Table S2 and levels of target mRNA were normalized to those of β-actin mRNA.

### 4.9. Statistical Analysis

The GraphPad Prism 8 software (GraphPad Software, San Diego, CA, USA) was used to perform statistical analyses. Statistical differences between two groups were analyzed using the Student’s *t*-test, whereas one-way analysis of variance (ANOVA) followed by Tukey–Kramer post hoc test or Kruskal–Wallis with Dunn’s multiple comparison was used to compare among multiple groups. A *p*-value < 0.05 was considered statistically significant.

## Figures and Tables

**Table 1:** Relative tissue weight (% of body weight) of WT, *SeBP1*-SK, and *SeBP1/2*-DK mice.

Tissue	Male (N = 9)			Female (N = 7)		
	WT	*SeBP1*-SK	*SeBP1/2*-DK	WT	*SeBP1*-SK	*SeBP1/2*-DK
Liver	4.73 ± 0.18	4.29 ± 0.19 *	4.18 ± 0.20 *	4.61 ± 0.11	4.48 ± 0.13	4.33 ± 0.12
Kidney	0.72 ± 0.04	0.69 ± 0.03	0.68 ± 0.03	0.69 ± 0.02	0.70 ± 0.02	0.72 ± 0.02
Lung	0.60 ± 0.01	0.61 ± 0.01	0.64 ± 0.01	0.76 ± 0.04	0.72 ± 0.02	0.70 ± 0.03
Heart	0.53 ± 0.03	0.50 ± 0.03	0.48 ± 0.03	0.57 ± 0.01	0.55 ± 0.02	0.55 ± 0.02
Brain	1.27 ± 0.01	1.26 ± 0.01	1.28 ± 0.02	1.75 ± 0.03	1.79 ± 0.04	1.78 ± 0.02
Spleen	0.37 ± 0.01	0.35 ± 0.01	0.34 ± 0.01	0.44 ± 0.02	0.43 ± 0.01	0.46 ± 0.01
Gonad ^a^	0.55 ± 0.01	0.54 ± 0.01	0.54 ± 0.01	0.02 ± 0.00 ^b^	0.02 ± 0.00 ^b^	0.02 ± 0.00 ^b^
eWAT	3.18 ± 0.02	3.21 ± 0.01	3.26 ± 0.01			

Each value represents the mean ± SEM. WT, wild type; *SeBP1*-SK, *SeBP1* knockout mice; *SeBP1/2*-DK, *SeBP1*, and *SeBP2* double-knockout mice. eWAT, epididymal white adipose tissue. Significant difference from WT: *, *p* < 0.05. ^a^ The weights of the testis (in males) and ovary (in females) were recorded. ^b^ The relative ovary weights (% of body weight) of WT, *SeBP1*-SK, and *SeBP1/2*-DK were 0.02 ± 0.003, 0.02 ± 0.001, and 0.02 ± 0.001, respectively.

## Data Availability

The raw data supporting the conclusions of this article will be made available by the authors on request.

## Supplementary Materials

The supporting information can be downloaded at: https://www.mdpi.com/article/10.3390/ijms26073363/s1.

## Abbreviations

The following abbreviations are used in this manuscript:

SeBP1	selenium-binding protein 1
SeBP2	selenium-binding protein 2
*SeBP1/2*-DK	*SeBP1* and *SeBP2* double-knockout mice
PPARA	peroxisome proliferator-activated receptor-alpha
hSeBP1	human selenium-binding protein 1
NEFA	non-esterified fatty acids
TG	triglyceride
HDL-C	high-density lipoprotein cholesterol
LDL-C	low-density lipoprotein cholesterol
TCDD	2,3,7,8-tetrachlorodibenzo-p-dioxin
*SeBP1*-SK	*SeBP1* single knockout mice
AST	aspartate aminotransferase
ALT	alanine aminotransferase
Rxra	retinoid X receptor alpha
Acox1	acyl-CoA oxidase 1
Acox3	acyl-CoA oxidase 3
Cpt1a	carnitine palmitoyl transferase 1A
Cpt2	carnitine palmitoyl transferase 2
Cyp4a12a	cytochrome P450, family 4, subfamily a, polypeptide 12A
Cyp4a12b	cytochrome P450, family 4, subfamily a, polypeptide 12B
Acaca	acetyl-coA carboxylase alpha 1
Fasn	fatty acid synthase
Srebp1	sterol regulatory element-binding protein 1
Pparg	peroxisome proliferator-activated receptor gamma
Hmgcr	3-hydroxy-3-methylglutaryl-CoA reductase
Srebp2	sterol regulatory element-binding protein 2
Lxra	liver X receptor alpha
Abca1	ATP-binding cassette transporter A1
Abcg1	ATP-binding cassette transporter G1
Abcg5	ATP-binding cassette transporter G5
Abcg8	ATP-binding cassette transporter G8
Npc1l1	Niemann–Pick c1-like 1
MDA	malondialdehyde
SOD	superoxide dismutase
H_2_O_2_	hydrogen peroxide
