# Supplementary material for: Ablation of Mouse Selenium-Binding Protein 1 and 2 Elevates LDL by Disruption of Cholesterol Efflux and Lipid Metabolism
Source: Int J Mol Sci. 2025 Apr 3;26(7):3363. doi: 10.3390/ijms26073363 (PMC11989624; doi:10.3390/ijms26073363)
Supplement: Supplementary file 1 [file ijms-26-03363-s001.zip › ijms-3523164-supplementary.pdf]

Supplemental Materials

Article

Ablation of mouse selenium-binding protein 1 and 2 elevates LDL by disruption of cholesterol efflux and lipid metabolism

Shuangli Zhao [1], Yingxia Song [1], Yuko Nakashima [1], Xing Zou [1], Takayuki Koga [2], Takumi Ishida [3], Renshi Li [4], Yuko Hirota [1], Yoshitaka Tanaka [1] and Yuji Ishii [1,*]

[1] Division of Pharmaceutical Cell Biology, Graduate School of Pharmaceutical Sciences, Kyushu University, Fukuoka 812-8582, Japan; zhao.shuangli.783@s.kyushu-u.ac.jp, songyingxia410@outlook.com, zouxing_hbtcm@163.com, yhirota@phar.kyushu-u.ac.jp, ytanaka@phar.kyushu-u.ac.jp, ishii@phar.kyushu-u.ac.jp

[2] Laboratory of Hygienic Chemistry, Daiichi University of Pharmacy, Fukuoka 815-8511, Japan; koga@daiichi-cps.ac.jp

[3] School of Pharmacy, International University of Health and Welfare Fukuoka, Ohkawa 831-8501, Japan; ishida@iuhw.ac.jp

[4] School of Traditional Chinese Pharmacy, China Pharmaceutical University, Nanjing 21198, China; li-renshi@cpu.edu.cn

[*] Correspondence: ishii@phar.kyushu-u.ac.jp, Tel.: (+81-92-642-6586)

Table S1. Sequences of primers used for genotyping

	Primer sequence	Product size
Genotyping-SeBP1-WT	Forward: 5′-TTGTTTCCCATCCACTGTCA-3′<br>Reverse: 5′-CATAGCTGAGTGTTGGGGGT-3′	829 bp
Genotyping-SeBP1-KO	Forward: 5′-GAACAAGATGGATTGCACGCAGGTTCTCCG-3′<br>Reverse: 5′-GTAGCCAACGCTATGTCCTGATAG-3′	668 bp
Genotyping-SeBP2-WT	Forward: 5′-ACAAAGTGTGGTCCAGGTTAC-3′<br>Reverse: 5′-TGGCTGGTGTGCGTATTG-3′	1297 bp
Genotyping-SeBP2-KO	Forward: 5′-CAGTCTTGACCAAATACACACACTC-3′<br>Reverse: 5′-TGTCCTACATACAGGAGTATTTTGC-3′	777 bp

Table S2. Sequences of primers used for PCR amplification of mRNAs

Target mRNA (Genebank Accession)	Primer sequence	Product size
SeBP1 (NM_009150)	Forward: 5′-CTGATACTGCCTGGTCTCA-3′<br>Reverse: 5′-AGTGGCTGGTGTGCAAAC-3′	142 bp
SeBP2 (NM_019414)	Forward: 5′-CTGATACTGCCTGGTCTCA-3′<br>Reverse: 5′-AGTGGCTGGTGTGCGTAT-3′	142 bp
Ppara (NM_011144)	Forward: 5′-CATCACAGACACCCTCTCTC-3′<br>Reverse: 5′-AAGCCCTTACAGCCTTCAC-3′	174 bp
Pparg (NM_011146)	Forward: 5′-AGACCACTCGCATTCCTTTGAC-3′<br>Reverse: 5′-TTTATCCCCACAGACTCGGCAC-3′	274 bp
Ppard (NM_011145)	Forward: 5′-AACACACGTTCCTTCCAG-3′<br>Reverse: 5′-GATCGCACTTCTCATACTCG-3′	237 bp
Rxra (NM_011305)	Forward: 5′-CTCCTTCACCAAGCACATC-3′<br>Reverse: 5′-GTCTTTGCGTACTGTCCTC-3′	115 bp
Cpt1a (NM_013495)	Forward: 5′-CTCCGCCTGAGCCATGAAG-3′<br>Forward: 5′-CACCAGTGATGATGCCATTCT-3′	100 bp
Cpt2 (NM_009949)	Forward: 5′-CCTGCTCGCTCAGGATAAACA-3′<br>Forward: 5′-GTGTCTTCAGAAACCGCACTG-3′	203 bp
Cyp4a12a (NM_177406)	Forward: 5′-GACTTCTATCACCTGGAATGAC-3′<br>Reverse: 5′-AGCTCTCTGCTCACACTTG-3′	105 bp
Cyp4a12b (NM_172306)	Forward: 5′-TACTCAGCAGTTCCCATCC-3′<br>Reverse: 5′-TCTCCCCAGAATCAGCTTC-3′	187 bp
Sod1 (NM_011434)	Forward: 5′-ATGGGTTCCACGTCCATCAG-3′<br>Reverse: 5′-GTCTCCAACATGCCTCTCTTC-3′	122 bp
Sod2 (NM_013671)	Forward: 5′-ACAACTCAGGTCGCTCTTC-3′<br>Reverse: 5′-ATAGCCTCCAGCAACTCTC-3′	128 bp
Acox1	Forward: 5′-TAACTTCCTCACTCGAAGCCA-3′	283 bp

(NM_001271898)	Reverse: 5′- AGTTCCATGACCCATCTCTGTC-3′	
Acox3 (NM_030721)	Forward: 5′-GGACAGGACTGGGAATATCAC-3′ Reverse: 5′-CAGACATGCTGATGATGGAG-3′	125 bp
Lxra (NM_001177730)	Forward: 5′-CTCAATGCCTGATGTTTCTCCT-3′ Forward: 5′-TCCAACCCTATCCCTAAAGCAA-3′	150 bp
Abcg1 (NM_009593)	Forward: 5′-GCTCCATCGTCTGTACCATCC-3′ Forward: 5′-ACGCATTGTCCTTGACTTAGG-3′	88 bp
Abca1 (NM_013454)	Forward: 5′-GCTTGTTGGCCTCAGTTAAGG-3′ Forward: 5′-GTAGCTCAGGCGTACAGAGAT-3′	135 bp
Abcg5 (NM_031884)	Forward: 5′-CGCGAGACGTTGCGATACA-3′ Forward: 5′-CTGCCAATCATTTGGTCCGC-3′	128 bp
Abcg8 (NM_001286005)	Forward: ATACCCTGGAGGTCTCATAGCA-3′ Forward: ACGTCGAGTAGTGAGGCTCTC-3′	134 bp
Hmgcr (NM_001360165.1)	Forward: 5′-CTGGAATTATGAGTGCCCCAAA-3′ Forward: 5′-ACGACTGTACTGAAGACAAAGC-3′	101 bp
Acaca (NM_133360.3)	Forward: 5′-ATGGGCGGAATGGTCTCTTTC-3′ Forward: 5′-TGGGGACCTTGTCTTCATCAT-3′	148 bp
Fasn (NM_007988.3)	Forward: 5′-GGAGGTGGTGATAGCCGGTAT-3′ Forward: 5′-TGGGTAATCCATAGAGCCCAG-3′	140 bp
Srebp1 (NM_011480.4)	Forward: 5′-GATGTGCGAACTGGACACAG-3′ Forward: 5′-CATAGGGGGCGTCAAACAG-3′	104 bp
Srebp2 (NM_033218.2)	Forward: 5′-GCAGCAACGGGACCATTCT-3′ Forward: 5′-CCCCATGACTAAGTCCTTCAACT-3′	200 bp
β-actin (NM_007393)	Forward: 5′-GATTACTGCTCTGGCTCCTA-3′ Reverse: 5′-TCCTGCTTGCTGATCCAC-3′	135 bp
hSeBP1 (NM_001258288)	Forward: 5′-TCCTCTACTTCAGCAACTGG-3′ Reverse: 5′-GTTTTCCCTTGACCACTAGG-3′	183 bp

PPARA	Forward: 5′-TTCGCAATCCATCGGCGAG-3′	146 bp
(NM_001001928)	Reverse: 5′-CCACAGGATAAGTCACCGAGG-3′	
ACOX1	Forward: 5′-GGAACTCACCTTCGAGGCTTG-3′	164 bp
(NM_001185039)	Reverse: 5′-TTCCCCTTAGTGATGAGCTGG-3′	
ACOX3	Forward: 5′-CGAGCTGAACTTCCTTCGATG-3′	107 bp
(NM_001101667.2)	Reverse: 5′- CCAGGCACTGAATCAAGGC-3′	
CPT1A	Forward: 5′-ATCAATCGGACTCTGGAAACGG-3′	121 bp
(NM_001031847)	Reverse: 5′-TCAGGGAGTAGCGCATGGT-3′	
SOD1	Forward: 5′-GGTGGGCCAAAGGATGAAGAG-3′	227 bp
(NM_000454)	Reverse: 5′- CCACAAGCCAAACGACTTCC-3′	
SOD2	Forward: 5′-GGAAGCCATCAAACGTGACTT-3′	116 bp
(NM_000636)	Reverse: 5′-CCCGTTCCTTATTGAAACCAAGC-3′	
β-ACTIN	Forward: 5′-GGCACCACACCTTCTACAATG-3′	163 bp
(NM_001101)	Reverse: 5′-AGCACAGCCTGGATAGCAAC-3′	

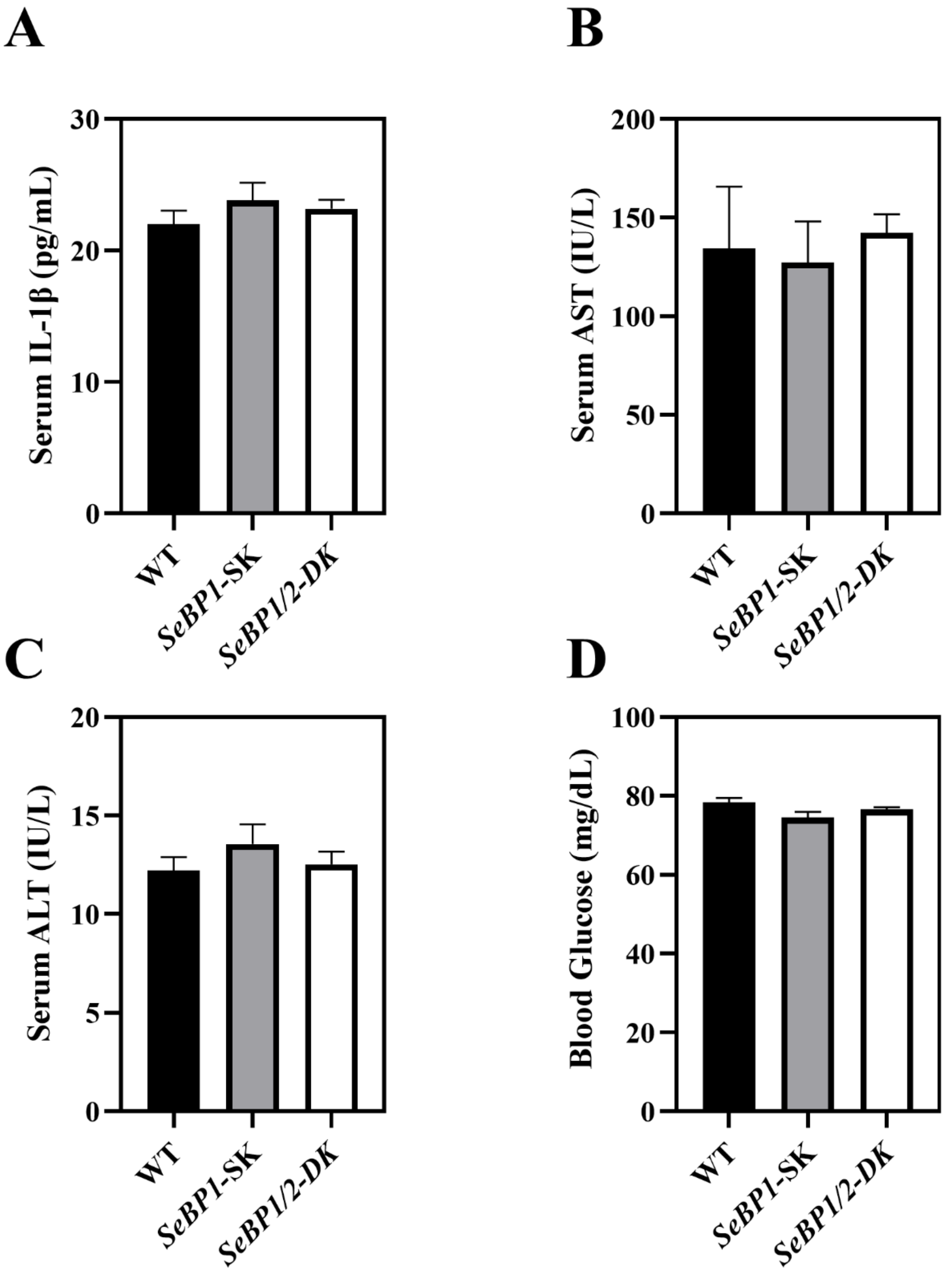

Figure S1. Effect of SeBP1/2 deletion on serum parameters. Each bar represents the mean ± SEM for 5 samples. IL-1β levels (A), serum AST (B), ALT(C) activity, and blood glucose level in WT, *SeBP1*-SK, and *SeBP1/2*-DK female mice were determined using commercially available kits at 8 weeks. WT, wild type; *SeBP1*-SK, *SeBP1* knockout mice; *SeBP1/2*-DK, *SeBP1* and *SeBP2* double-knockout mice.

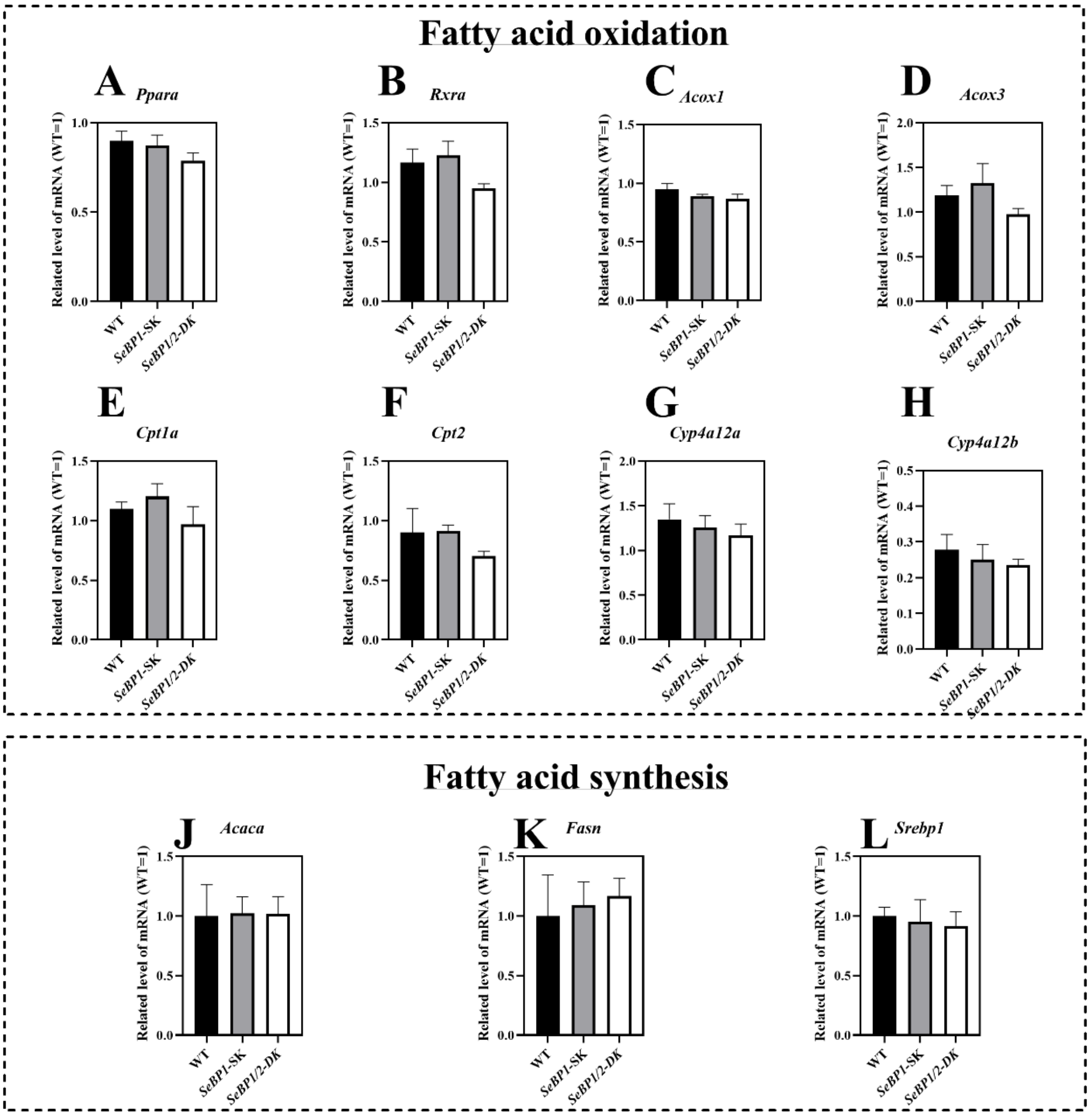

Figure S2. Key genes associated with β-oxidation of fatty acids in the liver. WT, *SeBP1*-SK, and *SeBP1/2*-DK female mice were fasted for 20 h at 8-weeks of age and euthanized by CO_2 inhalation. The livers were snap-frozen and stored at −80°C. Total RNA was isolated from the liver using a RNeasy Mini Kit (Qiagen, GmbH, Hilden, Germany). Values represent the means ± SEM for 5 mice. *Ppara*, peroxisome proliferator-activated receptor alpha; *Rxra*, retinoid X receptor alpha; *Acox1*, acyl-CoA oxidase 1; *Acox3*, acyl-CoA oxidase 3; *Cpt1a*, carnitine palmitoyltransferase 1A; *Cpt2*, carnitine palmitoyltransferase 2; *Cyp4a12a*, cytochrome P450, family 4, subfamily a, polypeptide 12A; *Cyp4a12b*, cytochrome P450, family 4, subfamily a, polypeptide 12B; *Acaca*, acetyl-coA carboxylase alpha; *Fasn*, fatty acid synthase; *Srebp1*, sterol regulatory element-binding protein 1; WT, wild type; *SeBP1*-SK, *SeBP1* knockout mice; *SeBP1/2*-DK, *SeBP1* and *SeBP2* double-knockout mice.

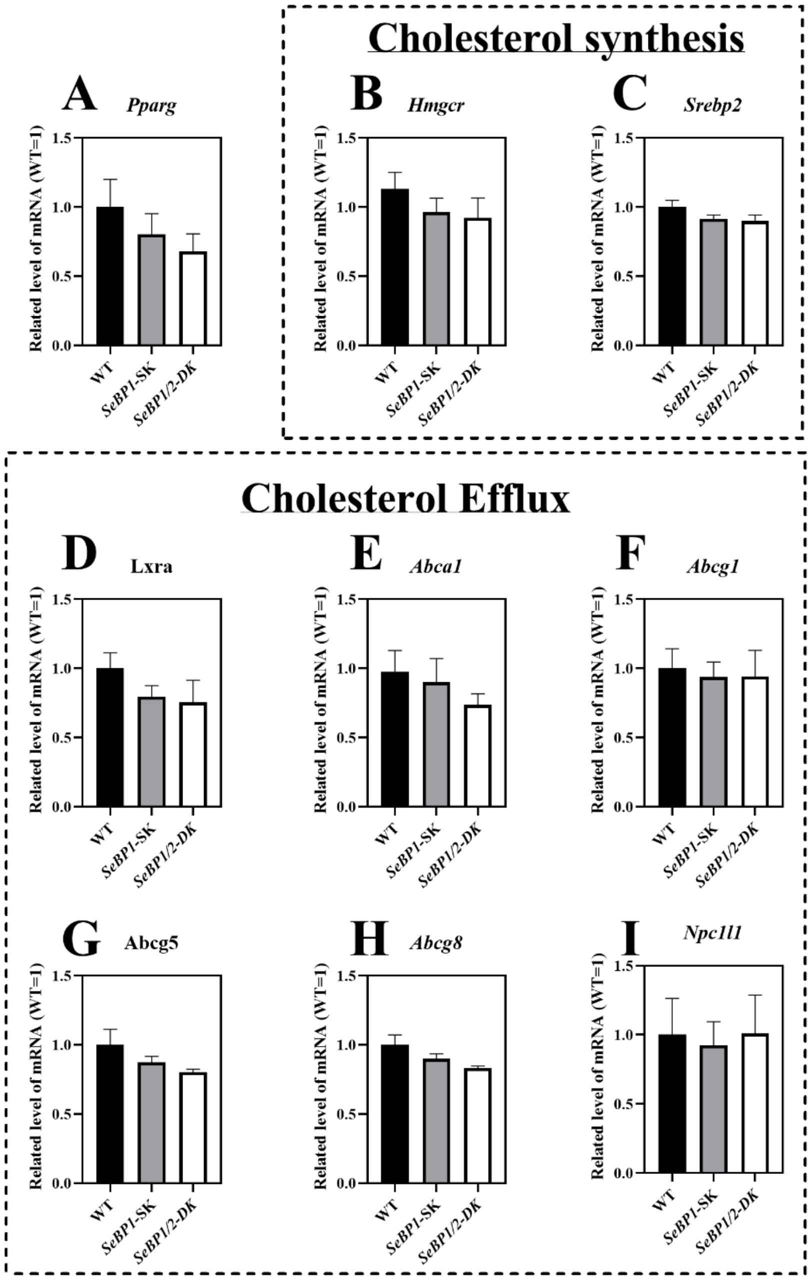

`

Figure S3. Key genes associated with cholesterol transport from the liver. WT, *SeBP1*-SK, and *SeBP1/2*-DK female mice were fasted for 20 h at 8-weeks of age and euthanized by CO_2 inhalation. The livers were snap-frozen and stored at −80°C. Total RNA was isolated from the liver using a RNeasy Mini Kit (Qiagen, GmbH, Hilden, Germany). Values represent the means ± SEM for 5 mice. *Pparg*, peroxisome proliferator-activated receptor gamma; *Hmgcr,* 3-hydroxy-3-methylglutaryl-CoA reductase; *Srebp2*, sterol regulatory element-binding protein 2; *Lxra*, liver X receptor alpha; *Abca1*, ATP-binding cassette transporter A1; *Abcg1*, ATP-binding cassette transporter G1; *Abcg5*, ATP-binding cassette transporter G5; *Abcg8*, ATP-binding cassette transporter G8; *Npc1l1*, niemann-pick c1-like 1; WT, wild type; *SeBP1*-SK, *SeBP1* knockout mice; *SeBP1/2*-DK, *SeBP1* and *SeBP2* double-knockout mice.

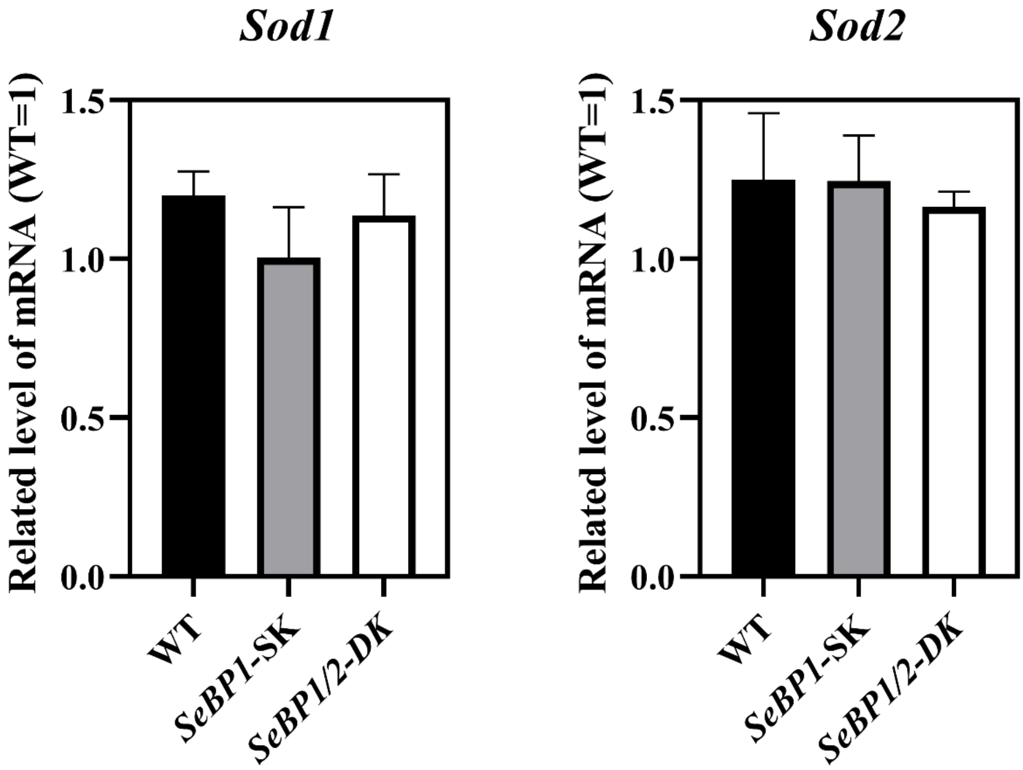

Figure S4. **Key genes associated with redox reaction in the liver.** WT, *SeBP1*-SK, and *SeBP1/2*-DK female mice were fasted for 20 h at 8-weeks of age and euthanized by CO_2 inhalation. The livers were snap-frozen and stored at −80°C. Total RNA was isolated from the liver using a RNeasy Mini Kit (Qiagen, GmbH, Hilden, Germany). Values represent the means ± SEM for 5 mice. *Sod1*, superoxide dismutase 1; *Sod2*, superoxide dismutase 2.
